# Antibacterial Properties of D-Amino Acid Oxidase: Impact on the Food Industry

**DOI:** 10.3389/fmicb.2019.02786

**Published:** 2019-12-03

**Authors:** Giorgia Letizia Marcone, Elisa Binda, Elena Rosini, Monica Abbondi, Loredano Pollegioni

**Affiliations:** ^1^Department of Biotechnology and Life Sciences, University of Insubria, Varese, Italy; ^2^D-Amino Acids International Reference Center, Gerenzano, Italy; ^3^Fondazione Istituto Insubrico Ricerca per la Vita, Gerenzano, Italy

**Keywords:** antibacterials, D-amino acids, D-amino acid oxidase, flavoenzymes, food safety, food preservation

## Abstract

Food quality is also related to safety and prevention of spoilage. Biological antimicrobial agents represent suitable alternatives to clinical preservatives in food industry to increase both safety and stability of aliments. Here, we focused on the enzyme D-amino acid oxidase (DAAO) from the yeast *Rhodotorula gracilis*, a well-studied protein for biotechnological use based on its stability, high activity, and easy recombinant production. DAAO catalyzes the O_2_-dependent oxidative deamination of D-enantiomer of amino acids generating α-keto acids, ammonia, and hydrogen peroxide. DAAO shows antibacterial activity on both Gram-positive and Gram-negative bacteria in the presence of D-alanine when tested on plates and reduced by half their growth when tested on liquid cultures. Control experiments performed with alternative amino acid-specific flavoenzymes (able or not to generate H_2_O_2_ acting on amino acids), a DAAO inactive variant, catalase (H_2_O_2_ scavenger), and L-amino acids instead of D-alanine identified H_2_O_2_ as the antibacterial agent. DAAO showed a good ability to decrease the bacterial growth on various food stuffs: e.g., 10-fold less colonies were formed on grated cheese incubated for 16 h at 37°C when a tiny amount (0.01 mg corresponding to 1.2 units) of DAAO was added. No exogenous D-amino acids were added since DAAO used the ones naturally occurring or the ones generated during ripening. Notably, simultaneously to H_2_O_2_ generation, DAAO also acts as O_2_-scavenger thus further hampering food deterioration.

## Introduction

The presence of bacterial pathogens in food may be responsible for spoilage and foodborne disease incidence. Furthermore, an increase of morbidity and mortality has been related to the emergence of multidrug resistant and disinfectant resistant bacteria (Vijayakumar and Sandle, 2019). Actually, preservative agents are added to ensure food safety and prevent spoilage. In this, both chemical and biological preservatives are used, for a review, see Brul and Coote (1999). Safety and stability of manufactured foods is gained by adding chemical preservatives such as weak acids, i.e., benzoic and sorbic acids (Brul and Coote, 1999): their use can cause microbiological resistance. Moreover, pathogenic bacteria, like *Listeria monocytogenes* cannot be fully eliminated in food products by chemical preservatives, which also do not delay the growth of spoilage microorganisms (Tajkarimi et al., 2010). On this side, the antibacterial effects of hydrogen peroxide have been extensively investigated due to its possible involvement in a number of important biological events in which bacterial cells are either killed or their growth inhibited. Hydrogen peroxide generates a short lined singlet O_2_ species, which is extremely biocidal, as well as superoxide radicals that in the presence of trace amounts of transition metal ions generate biocidal hydroxyl radicals. Hydrogen peroxide has a potential to be used in a variety of ways in the food industry as antimicrobial agent in water and dairy products (Juven and Pierson, 1996).

In recent years, interest focused on the use of natural antimicrobial agents in foods, such as antimicrobial peptides and occurring proteins, e.g., lysozyme, lactoperoxidase, or lactoferrin. In the food industry, aerobic microorganisms are affected by glucose oxidase-catalase system, which acts by depleting available oxygen and generating hydrogen peroxide (Wong et al., 2008).

D-Amino acid oxidase (EC 1.4.3.3, DAAO) is a dimeric enzyme containing one molecule of FAD per 40 kDa monomer. It belongs to the dehydrogenase/oxidase class of flavoproteins that catalyze with a strict stereospecificity, the oxidative deamination of D-amino acids to give α-keto acids and ammonia: reoxidation of reduced flavin by O_2_ generates hydrogen peroxide (Pollegioni et al., 2007). Microbial DAAOs (especially the ones from the yeast *Rhodotorula gracilis* and *Trigonopsis variabilis*) possess properties compatible with industrial applications: e.g., high activity on a number of neutral and polar D-amino acids, a strong interaction with the cofactor and a good stability (Pilone et al., 1989; Pollegioni et al., 1993, 2008; Molla et al., 2000; Pilone and Pollegioni, 2002). Accordingly, yeast DAAO is used in the production of 7-amino cephalosporanic acid from cephalosporin C, in the resolution of natural and synthetic racemic mixtures of amino acids and for the detection and quantification of D-amino acids in biological samples and foodstuff (a good parameter of bacterial contamination or aging) (Pollegioni and Molla, 2011).

Here, we report on the antibacterial activity of DAAO from *Rhodotorula gracilis*: its reaction uses O_2_ and generates hydrogen peroxide, a trait used in food preservation.

## Materials and Methods

### Materials

All chemical reagents, including media, antibiotics, and catalase, were purchased from Sigma-Aldrich, Milan, Italy. All the chemical reagents were used without additional purification.

### Bacterial Strains and Growth Conditions

*Escherichia coli* ATCC 35218, *Bacillus subtilis* ATCC 6633, *Pseudomonas aeruginosa* ATCC 10145, *Salmonella enterica subsp. typhimurium* ATCC 6994, and *Staphylococcus aureus* ATCC 6538P (methicillin susceptible *S. aureus*, MSSA) were obtained from the American Type Culture Collection (ATCC). *Acinetobacter baumannii*, *Enterococcus faecalis*, and *Yersinia enterocolitica* were clinical isolates. *E. coli*, *B. subtilis*, *P. aeruginosa*, *S. enterica* subsp. *typhimurium*, *E. faecalis*, *Y. enterocolitica*, and *A. baumannii* were propagated overnight in Luria Bertani medium (LB, 2% tryptone, 2% yeast extract, and 1% NaCl). *S. aureus* in Mueller Hinton broth 2 (MHB2, 0.3% beef infusion solids, 1.75% casein hydrolysate, and 0.15% starch) with continuous shaking at 200 rpm and 37°C. For exponential growth, overnight cultures were transferred to fresh medium: start cultures showed an optical density at 600 nm (OD_600 nm_) of 0.1. Storage at −20°C in 20% glycerol was used for long-term preservation.

### Enzymes

Recombinant *Rhodotorula gracilis* DAAO wild-type was produced in *E. coli* cells and purified with a 95% of purity as stated in Fantinato et al. (2001). Recombinant DAAO variants R285A and mDAAO were produced in *E. coli* cells and purified both with a 95% purity as stated in Molla et al. (2000) and Rosini et al. (2009). Recombinant D-aspartate oxidase (DASPO) and L-amino acid oxidase (LAAD) were produced in *E. coli* cells and purified with a 95 and 90% purity, respectively, as stated in Motta et al. (2016) and Molla et al. (2019). The specific activity of DAAO wild-type, R285A, and mDAAO are 120, 0.01, and 120 U/mg, respectively. DASPO and LAAD show a specific activity of 95 and 3.8 U/mg, respectively. Catalase was purchased from Sigma-Aldrich (Milan, Italy): specific activity 10,000 U/mg protein.

### Agar Diffusion Test

Antibacterial activity of the different enzymes was evaluated against *E. coli* ATCC 35218, *B. subtilis* ATCC 6633, and *S. aureus* ATCC 6538P by agar diffusion assay (Finn, 1959). Fresh bacterial cultures, inoculated from overnight pre-cultures, were grown in LB or MHB2 medium until an OD_600 nm_ = 0.4 and then used to prepare agar plates containing Antibiotic Medium 1 (AM1) or Mueller-Hinton Agar (MHA) medium with or without adding increasing concentrations (0.2, 2, 10 mM) of D-alanine (or D-aspartate for DASPO) or 0.2, 2, 10, 20 and 40 of D,L-alanine. A drop of 10 μl containing increasing concentrations (10, 100, 1,000 μg/ml) of enzymes (wild-type, R285A variant, and mDAAO, DASPO or LAAD) were loaded onto the inoculated plates and then incubated at 37°C for 24 h. The diameter of bacterial growth inhibition zone surrounding the site of drop deposition was measured.

The effect of catalase on the antibacterial activity of DAAO enzymes was tested on the same strains. Briefly, 10 μl of catalase at 1 mg/ml concentration were added to inoculated AM1 or MHA plates with or without 10 mM of D-alanine together with 10 μl of the enzymes at 1 mg/ml concentration.

### Determination of Minimal Inhibitory Concentration (MICs)

Cultures of *E. coli* ATCC 35218, *A. baumannii*, *P. aeruginosa* ATCC 10145, *S. enterica* subsp. *typhimurium* ATCC 6994, *E. faecalis*, *Y. enterocolitica*, *B. subtilis* ATCC 6633, and *S. aureus* ATCC 6538P were treated as follows to determine the MICs of DAAO. Cryovials of glycerinates were thawed at room temperature and used to inoculate LB or MHB2 media. The strains were grown to exponential growth phase (~OD_600nm_ = 0.4) at 37°C with shaking at 200 rpm. Then, 10 μl of cultures were seeded onto LB or MH agar plates supplemented with 10 mM D-alanine and increasing concentrations of DAAO: from 0 to 100 μg/ml in 10 μg/ml increments. Following drying, plates were incubated at 37°C. The MIC values represent the lowest DAAO concentration that inhibited visible growth after 24 h of incubation.

### Liquid Growth Kinetics

Growth kinetics of liquid cultures of *B. subtilis* ATCC 6633, *S. aureus* ATCC 6538P, and *E. coli* ATCC 35218 were recorded by measuring the OD_590nm_ using an Infinite® 200 spectrophotometer (TECAN, Milan) at regular time intervals. Preinocula were prepared from cultures in LB or MHB2 medium grown overnight (at 37°C and at 200 rpm). Experiments were carried out in 96 well plates: each well, containing 200 μl of LB or MHB2 medium, was added of 10 μg of DAAO (corresponding to 1.2 units) and 10 mM substrate.

### Bactericidal Effect of D-Amino Acid Oxidase on Grated Cheese

A total of 10 g of a commercial grated cheese (mix of Grana Padano and Parmigiano Reggiano 12 months ripened, previously sterilized by UV irradiation to eliminate the innate onset of bacteria) were left in Petri dishes and incubated at room temperature for a maximum of 168 h. Every 24 h, the cheese sample from one plate was processed as reported in Rosini et al. (2008). Briefly, the fat part was removed by centrifugation and the water extract was plated on LB agar with or without adding 0.4% (w/v) DAAO (1 mg/ml, corresponding to 2.4 units per plate). After 16 h of incubation at 37°C, microbial colonies were counted and expressed as colonies forming unit (CFU)/g grated cheese.

### Bacteriological Analysis and Total D-Amino Acids Content of Food Samples

Samples of parmesan, different baby food (fruit, turkey, and fish), and raw chicken breast meat fillets were provided by local supermarket. Cheese, baby food, and meat samples were divided into portions of 1 g each and left in sterile Petri dishes with 0.4% (w/v) DAAO (1 mg/ml). The control samples were similarly prepared, except for adding DAAO. Each condition was tested in triplicate. Petri dishes were stored at 6 ± 1°C for 15 days and then analyzed for microbial counts.

Each sample was diluted [10% (w/v)] in buffered peptone water and mixed for 2 min using a vortex. The suspension of parmesan was treated as reported in Rosini et al. (2008), see above. The suspension of other food specimen was 10-fold serially diluted in the buffered peptone water. An aliquot of 10 μl of each sample was subsequently plated in triplicate on different media (Mc Conkey, Brilliant Green and MHA). Plates were examined visually for colony type and morphological characteristics based on the selective growth medium used. After incubation for 24–48 h at 37°C, colonies were counted and expressed as log_10_ colonies forming unit (CFU)/g food sample.

Total D-amino acid content in food samples was assayed using a fluorescence-based biosensor made of two cuvettes for fluorescence analysis containing the Nile Red fluorescence dye and a mixture of M213G and T60A/Q144R/K152E DAAO variants (0.5 μM each), respectively (Rosini et al., 2008, 2014). A calibration curve was obtained using a standard D-alanine solution (in the 0–25 μM concentration range, *n* = 3). The fluorescence emission values were recorded at 623 nm with excitation at 450 nm, using a Jasco FP-750 spectrofluorimeter (Jasco, Cremello, Italy), at room temperature. The detection limit was 0.1 μM, with a limit of quantification of 0.22 μM. D-amino acids solutions (5 μM final concentration) containing different ratios of D-alanine (0–5.0 μM), D-glutamate (0–4.0 μM), D-lysine (0–4.0 μM), D-glutamine (0–1.7 μM), and D-methionine (0–1.0 μM) were used to evaluate the effect of the substrate composition on the biosensor response; a mean relative fluorescence value corresponding to ≈70% of the value obtained on 5 μM D-alanine was measured. Homogenized samples and the chicken breast meat fillet were suspended in 100 mM disodium pyrophosphate buffer, pH 8.5 at a final concentration of 0.1 g/ml and incubated for 15 min at 20°C in an ultrasonic bath. The suspension was centrifuged at 11,000*g* for 30 min at 4°C, and the supernatant was used for biosensor measurements.

## Results

### Antibacterial Activity of D-Amino Acid Oxidase: Plate Assay

**Figure 1:**
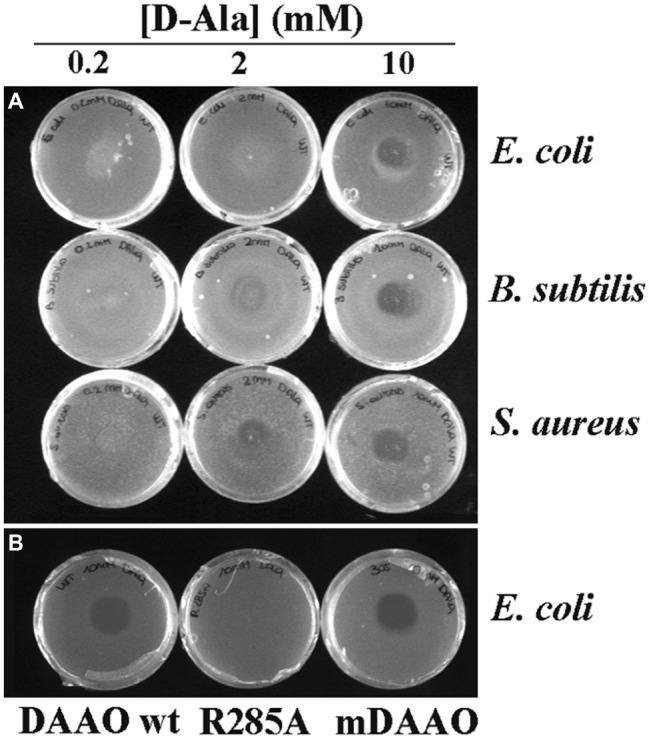
Antibacterial activity of DAAO. **(A)** Plates of *E. coli* ATCC 35218 (top), *B. subtilis* ATCC 6633 (middle), and *S. aureus* ATCC 6538P (bottom) incubated with different concentrations of D-Ala (0.2 mM on the left, 2 mM on the center, and 10 mM on the right) using 0.01 mg of DAAO corresponding to 1.2 units per plate. **(B)** Plates of *E. coli* ATCC 35218 incubated with 10 mM D-Ala and 0.01 mg of different variants of DAAO: wild-type (left), R285A (middle) and mDAAO (right).

At first, the antibacterial activity of DAAO was investigated using an agar plate diffusion assay by comparing the growth inhibitory effects on two commonly used Gram-positive bacteria, i.e., *S. aureus* ATCC 6538P and *B. subtilis* ATCC 6633, and on the Gram-negative bacteria *E. coli* ATCC 35218. Assays were carried out with or without adding the DAAO substrate in the plates (i.e., 0.2, 2, and 10 mM D-alanine or 0.2, 2, 10, 20, and 40 mM of D,L-alanine). Wild-type DAAO inhibited the growth of all the strains tested only when D-alanine was present in the medium: L-alanine at the same concentrations did not allow the formation of inhibition halos, as expected since L-amino acids are not substrates of DAAO (data not shown) (Pollegioni et al., 2008). The halos were more clearly visible using 10 mM of D-alanine: accordingly, this concentration was used in the following tests. The best condition in terms of halo size (10 mm) was observed using 10 mM of D-alanine or 20 mM D,L-alanine (indicating that the racemic amino acid mixture can be also used) and 0.01 mg of DAAO corresponding to 1.2 units (per plate, Figure 1A). The same assay was also performed using an identical amount of R285A (an inactive variant, corresponding to 0.0001 units) or mDAAO (a variant also active at low O_2_ concentration based on a ~10-fold lower K_m_ for dioxygen, corresponding to 1.2 units at air O_2_ saturation) DAAO variants: the R285A variant did not produce any inhibition halos, while the halos generated by mDAAO were comparable (11 mm vs. 10 mm, respectively) to those produced by DAAO wild-type (Figure 1B).

We then examined the minimal bactericidal activity of DAAO against *E. coli* ATCC 35218, *A. baumannii*, *P. aeruginosa* ATCC10145, *S. enterica* subsp. *typhimurium* ATCC 6994, *E. faecalis*, *Y. enterocolitica*, *B. subtilis* ATCC 6633, and *S. aureus* ATCC 6538P. All bacteria were incubated with DAAO at various concentrations in the presence of 10 mM D-alanine in LB or MH agar medium. A total of 10, 20, and 25 μg/ml of DAAO yielded *E. faecalis, Y. enterocolitica*, and *S. aureus* growth inhibition, respectively. The minimal concentration of DAAO required to inhibit *E. coli* and *B. subtilis* growth was 50 μg/ml, while to inhibit *A. baumannii* and *P. aeruginosa* growth was 60 and 70 μg/ml, respectively. Notably, *S. enterica* subsp. *typhimurium* growth was not affected by the DAAO-D-alanine treatment.

### Identification of the Antibacterial Agent

**Figure 2:**
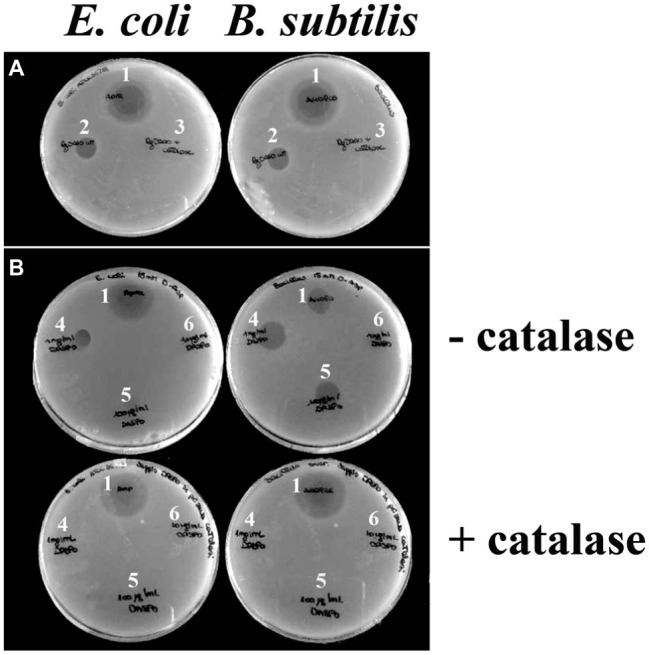
Effect of catalase on DAAO and DASPO antibacterial activity. Plates containing *E. coli* ATCC 35218 (left) and plates containing *B. subtilis* ATCC 6633 (right). **(A)** For both plates, it is possible to appreciate an halo due to an antibiotic (as positive control, 1) and an halo due to DAAO (2); no inhibition halo was apparent where the mixture DAAO-catalase was loaded (3). **(B)** In top panels, where no catalase is present, it is possible to appreciate inhibition halos with different sizes depending on the quantity of DASPO enzyme loaded 10 μl of [(4) 1,000 μg/ml; (5) 100 μg/ml; (6) 10 μg/ml]. In both cases, the wider halo is the one where 10 μg of enzyme were plated (4). In the presence of catalase (bottom panels), halo is observed only where the antibiotic was loaded (1).

To prove that the antibacterial effect is due to the production of hydrogen peroxide by DAAO, the assay was carried out on plates loading 0.01 mg of wild-type DAAO (approx. 1.2 unit) with or without adding the same amount of catalase, an enzyme that eliminates the hydrogen peroxide produced by DAAO (because of the high specific activity of catalase, the used amount corresponds to a 5,000-fold excess compared to DAAO in terms of enzymatic units). In the presence of catalase, no bactericidal effect is observed, thus strongly supporting hydrogen peroxide as the antibacterial agent (Figure 2A).

To confirm the proposal that the antibacterial activity of DAAO is mainly due to hydrogen peroxide generation during the reaction with D-alanine present in the medium, we used an alternative flavoenzyme also able to efficiently generate hydrogen peroxide such as D-aspartate oxidase (DASPO). To this purpose, *E. coli* and *B. subtilis* were incorporated into agar plates containing 15 mM of D-aspartate (the best substrate of DASPO) (Katane et al., 2007) and added of 10 μl of DASPO at different concentrations (10, 100, and 1,000 μg/ml corresponding to ~0.0095, 0.095, and 0.95 units, respectively) with or without adding the catalase, see above. As shown in Figure 2B, in the absence of catalase the wider inhibition halo (13 mm) is formed in the presence of the highest amount of DASPO (10 μg per plate). No halos are visible in the presence of catalase.

To further endorse the fact that the antibacterial activity of DAAO is due to the production of hydrogen peroxide, a diffusion agar test was done using a plate containing 10 mM of l-phenylalanine and loading different quantities of L-amino acid deaminase (LAAD). This flavoenzyme, a member of amino acid oxidase family, deaminates L-amino acids with no hydrogen peroxide production (Motta et al., 2016). In this case, no halos are observed in all the conditions tested (data not shown).

### Antibacterial Activity of D-Amino Acid Oxidase: Liquid Culture

**Figure 3:**
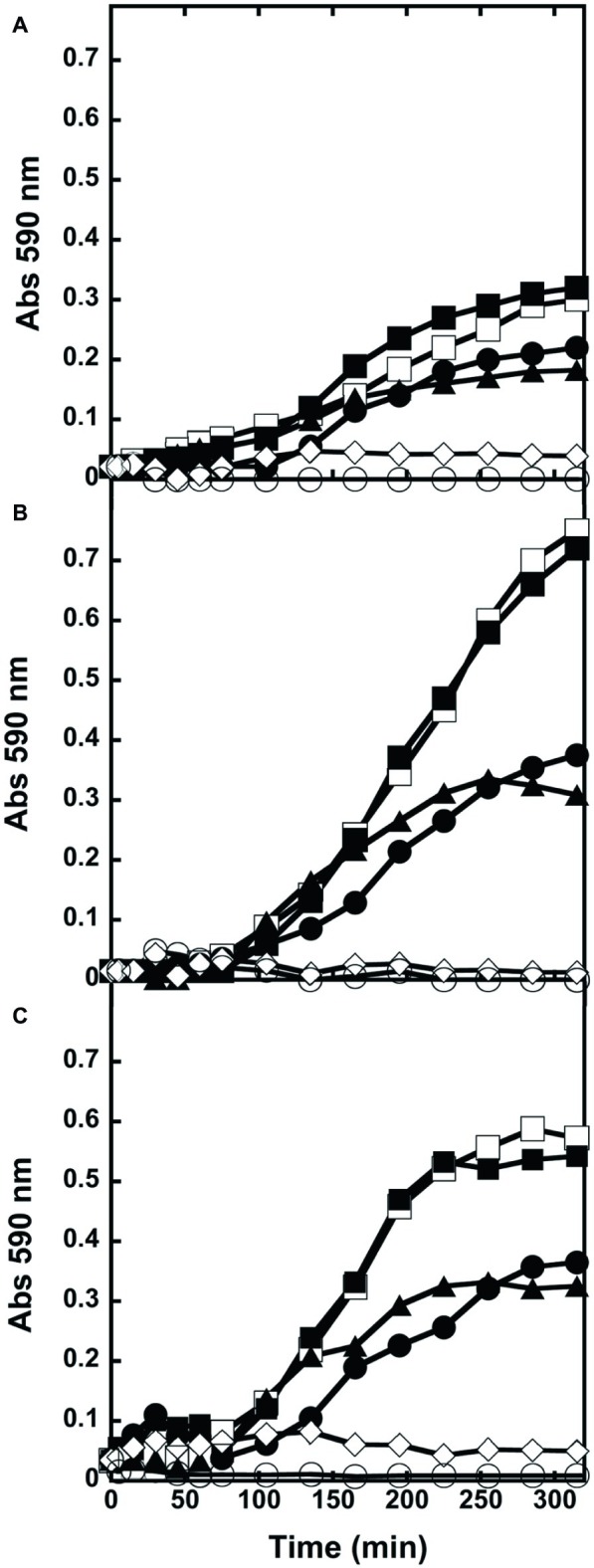
Kinetics of growth of liquid cultures. **(A)** *E. coli* ATCC 35218, **(B)** *B. subtilis* ATCC 6633, and **(C)** *S. aureus* ATCC 6538P exposed to wild-type (●), R285A (■), mDAAO (▲), antibiotic (◊) or hydrogen peroxide (○). Cultures without any addition (□) were used as controls. Growth was recorded for 5 h. Triplicate experiments were conducted for each condition: standard errors were lower than 5%.

We further investigated the effect of wild-type DAAO and its R285A and mDAAO variants, on bacterial viability by adding the enzymes at the log phase of growth of *E. coli* ATCC 35218 (Figure 3A), *B. subtilis* ATCC 6633 (Figure 3B), and *S. aureus* ATCC 6538P (Figure 3C) cultivations. Cultures with no added enzymes or to which antibiotics or hydrogen peroxide were added have been used as negative and positive controls, respectively. Figure 3 shows that the three strains equally responded to the enzymes’ addition. As expected, the growth kinetics were dramatically affected by hydrogen peroxide or ampicillin (for *E. coli*) or teicoplanin (for Gram-positive bacteria): albeit with a slightly different kinetics, cell density was drastically reduced after 5 h of incubation. Indeed, the active wild-type and mDAAO variant (differing in O_2_ affinity) similarly reduced by half the growth of the three strains. This result indicates that DAAO antibacterial activity is not affected by O_2_ concentration under the tested conditions. Finally, the R285A DAAO variant (inactive) did not affect growth of the tested strains.

### Antibacterial Effect of D-Amino Acid Oxidase on Foods

**Figure 4:**
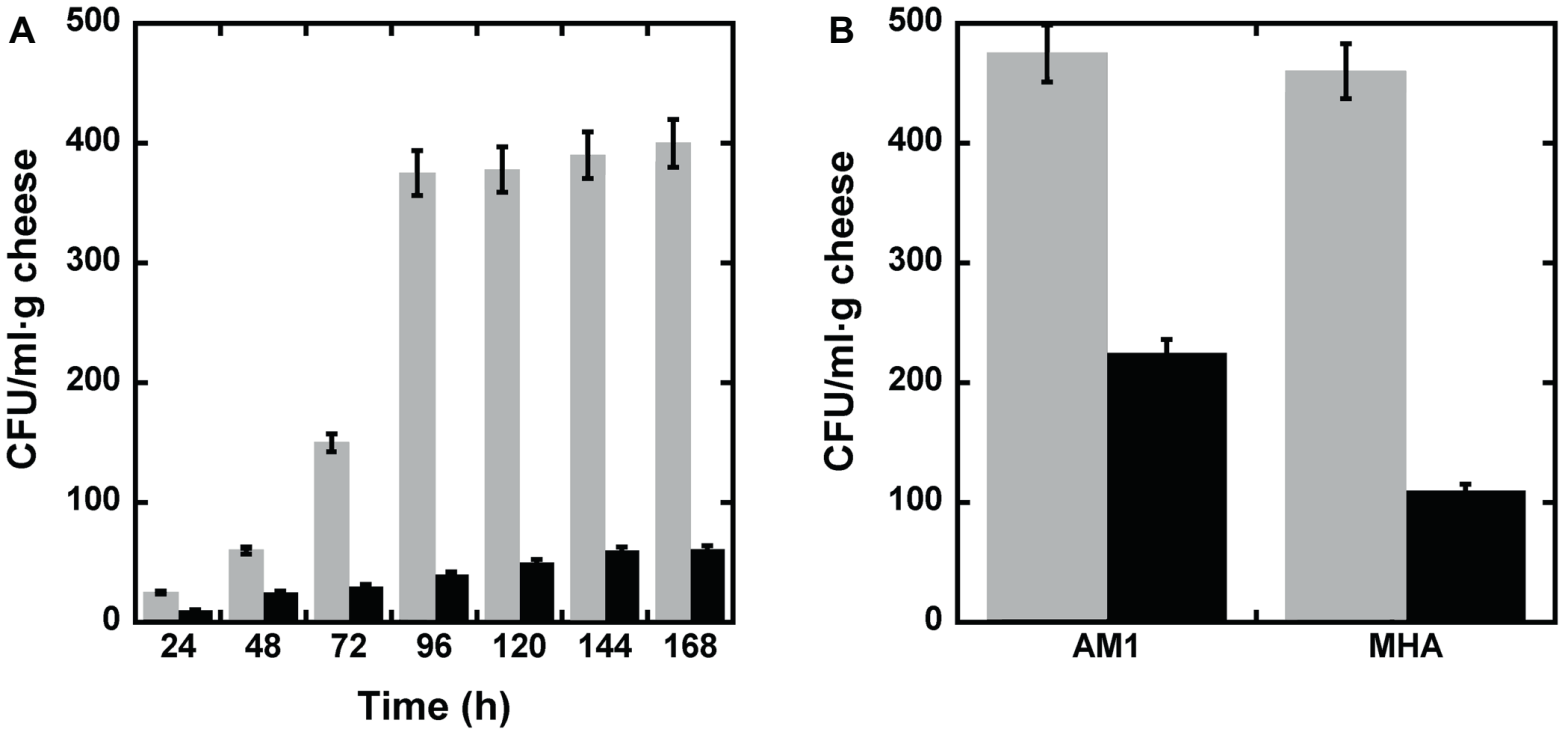
Bactericidal activity of DAAO. **(A)** Bacterial cell viability measured, every 24 h of incubation, as CFU/ml·g of grated cheese in the presence (black bars) or absence (untreated control, light gray bars) of DAAO. **(B)** Bacterial cell viability measured after 15 days of incubation, as CFU/ml·g of parmesan in the presence (black bar) or absence (untreated control, light gray bars) of DAAO.

To check whether DAAO is able to reduce bacterial contamination on food samples, seven samples containing 10 g of grated cheese each (i.e., a commercial mixture of different cheeses) were analyzed following the protocol of Rosini et al. (2008) to remove the fat component of cheese: 100 μl of supernatant were plated with or without adding 0.01 mg of wild-type DAAO (corresponding to 1.2 units). After 16 h of incubation at 37°C, CFU were counted. As shown in Figure 4A, when no enzyme was added to the supernatant approximately 10 times more colonies were counted than in plates spread with supernatant plus DAAO. This result demonstrates once more the antimicrobial activity of DAAO enzyme.

Subsequently, the same experiment was performed on slices of parmesan. For this purpose, a slice of parmesan (1 g) was spread with 0.4% (w/w) DAAO (2.4 units); a slice of untreated parmesan was used as control. The cheese’s slices were conserved into Petri dishes in the fridge (6°C) for 15 days. At the end of the incubation period, the parmesan was processed as reported in section “Materials and methods” (section “Bactericidal Effect of D-Amino Acid Oxidase on Grated Cheese”) and the supernatants plated on two different selective media (AM1 or MHA to promote Gram-positive or Gram-negative bacteria growth, respectively). As shown in Figure 4B, in both media a higher concentration of bacteria was detectable in the slices not containing DAAO than in DAAO containing samples. This result allows to propose the use of DAAO as preservative agent for cheeses.

**Figure 5:**
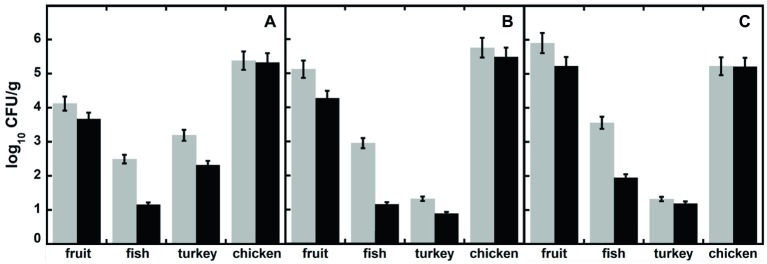
Total viable bacteria (expressed as log_10_ CFU/g of food), in different food samples measured after 15 days of incubation, in the presence (black bars) or absence (untreated control, light gray bars) of DAAO. Samples were plated in different media: **(A)** Mc Conkey agar, **(B)** Brilliant Green, and **(C)** Mueller Hinton agar.

In order to verify the effectiveness of DAAO as biopreservative agent, four additional food samples (i.e., homogenates of fruit, fish, turkey, and chicken breast meat fillets) were conserved at 6°C in sterile Petri’s dishes with or without adding DAAO [0.4% (w/w) corresponding to 2 U/g of food]. After 2 weeks, all the samples were diluted [10% (w/v)] in buffered peptone water as enrichment media, and then 0.01 ml of 10-fold diluted suspension was plated in triplicate on different selective media (Mc Conkey, Brilliant Green and MHA). After 24–48 h at 37°C, plates were observed for morphology and counted for the total number of CFU (see Figure 5 where data are expressed as log_10_ CFU/g food sample). As a general rule, the plates containing DAAO-treated samples show a lower development of colonies respect to plates with samples incubated without DAAO. In the plates containing agar Mc Conkey medium, selective for Gram-negative bacteria, several pink to red colonies were observed indicative of lactose-fermenting organisms, such as *E. coli* and *Klebsiella* spp.: only few colorless or clear colonies were apparent, indicating lactose-non-fermenting organisms, such as *Salmonella*, *Shigella*, and *Proteus* spp. No red-pink-white opaque colored colonies surrounded by brilliant red zones were found in plates containing Brilliant Green agar medium (indicating the bacteria belonging to *Salmonella* genus), neither small red colonies (indicating absence of *Proteus* and *Pseudomonas* species).

In these treatments, the DAAO antibacterial activity is based on the use of D-amino acids present in the foodstuffs: actually, no effect was observed for the chicken sample, which does not contain D-amino acids (Table 1). The total D-amino acids concentration in the food samples does not correspond to the observed inhibition of bacterial growth: the highest antibacterial effect was observed for the fish sample, while the highest D-amino acid level was apparent in the fruit one (Table 1). This observation indicates that the D-amino acid composition and the original bacterial composition in the different food samples affect the DAAO antibacterial activity. Such an effect is probably related to the ripening food process: over the time, some bacteria can release D-amino acids in the media (Genchi, 2017).

**Table 1:** Amount of total D-amino acids in different foods and average bacterial growth inhibition induced by DAAO treatment (2 U/g food).

	D-Amino acids		Bacterial inhibition
	(mM)	(mg/g food)	(%)
Cheese	10.5 ± 1.5	7.2 ± 1.0	85
Fruit	1.4 ± 0.2	1.6 ± 0.2	58
Fish	0.2 ± 0.01	0.2 ± 0.01	97
Turkey	0.9 ± 0.1	1.1 ± 0.1	65
Chicken	b.d.	b.d.	16

*b.d., below detection*.

## Discussion

The antimicrobial activity of DAAO was first reported by Cline and Lehrer in 1969 related to its putative physiological role in leukocytes (Cline and Lehrer, 1969), followed by a couple of additional studies (Zhang et al., 2004; Nakamura et al., 2012). To the best of our knowledge, this is the first report on using DAAO as an antibacterial agent applied at foodstuffs. We selected DAAO from the yeast *Rhodotorula gracilis* for this application since it can be overexpressed in huge amounts in *E. coli* cells (up to 100 mg/l fermentation broth and at low cost, 0.04 €/enzyme unit) (Romano et al., 2009); it shows a strong interaction with the FAD cofactor (i.e., it is always present in solution as active holoenzyme) (Pollegioni et al., 2007), a high kinetic efficiency (maximal activity at air O_2_-saturation is >100 U/mg protein), a broad substrate preference for D-amino acids (only acidic D-amino acids are not oxidized), and absence of inhibition by the L-enantiomer. Because of the latter property, racemic mixtures of amino acids can be used instead of pure D-amino acid solutions, resulting in a lower cost of the assay.

In the present work, we tested the antibacterial activity of DAAO by diffusion agar test and liquid growth kinetics: in both cases, we demonstrated that active wild-type DAAO and its variant mDAAO are able to inhibit bacterial growth producing an inhibition halo on cultures grown on agar medium and to reduce by half the growth in liquid culture. Furthermore, the antibacterial activity was proved against both Gram-positive (*B. subtilis* and *S. aureus*) and Gram-negative (*E. coli*) bacteria. In order to verify that the DAAO antibacterial activity is due to hydrogen peroxide production, we compared the DAAO effect with that of two related amino acid oxidases: DASPO, a hydrogen peroxide producing flavoenzyme, behaved similarly to DAAO, while LAAD, which is known to transfer electrons from reduced FAD to a cytochrome b-like protein with no H_2_O_2_ production (Motta et al., 2016), did not show any antibacterial activity. Indeed, both DAAO and DASPO in the presence of catalase, which catalyzes the decomposition of hydrogen peroxide to water and oxygen, lost the antibacterial activity. We conclude that the antibacterial activity of yeast DAAO is due to the production of hydrogen peroxide, thus confirming the conclusions from a previous study based on porcine DAAO (Nakamura et al., 2012). Our finding also agrees with *in vivo* results. Actually, the endogenous expression of DAAO in kidney was considered sufficient to reduce bacterial growth (Nakamura et al., 2012), and DAAO was reported to bind to bacterial cell walls, yielding a more localized and concentrated production of H_2_O_2_ (Zhang et al., 2004). DAAO showed antibacterial activity on seven out of the eight bacterial strains used: *S. enterica* subsp. *typhimurium* was not affected by the DAAO-D-alanine antibacterial treatment. This is an intriguing issue since *Salmonella* is known to evade the oxidative damage elicited by DAAO reaction in neutrophils by expressing an ABC importer specific for D-alanine (Tuinema et al., 2014).

In this work, DAAO has been evaluated for possible use in food preservation. In food samples, the development of bacteria on the plates was significantly reduced by treatment with DAAO in comparison to the untreated controls. Bacterial growth in grated cheese is reduced 10-fold by adding DAAO, as well as the bacterial level in 2-weeks aged foodstuffs. Notably, in D-amino acid-rich foodstuffs, no exogenous addition of D-amino acids is required to appreciate the antibacterial activity of DAAO.

The overall reaction catalyzed by DAAO involves the consumption of one D-amino acid and one oxygen molecule to produce one α-keto acid, one ammonia, and one hydrogen peroxide molecule. This reaction uses O_2_, a property that could allow DAAO to be used as an active O_2_-scavenger, antioxidant, and preservative in food applications. For example, lipid oxidation can result in deterioration and rancid taste in high-fat foods, i.e., mayonnaise (Isaksen and Adler-Nissen, 1997). In canned/bottled/packaged food, oxygen favors bacterial growth: O_2_ removal from the headspace helps to maintain an anaerobic environment (Kirk et al., 2002) and to preserve taste and flavor of beverages such as wine and beer (Labuza and Breene, 1989; Dube et al., 2017). Glucose oxidase is typically used as food processing-additive in a mixture with catalase, since the two enzymes are present together in the mycelium cell wall of fungi and enzyme isolation is costly: while this system works well in O_2_ scavenging, the antibacterial activity is limited by deactivation of hydrogen peroxide (Wong et al., 2008). On the contrary, based on the low amount required and the low cost of production, DAAO is used as purified preparation: this allows both efficient hydrogen peroxide generation and O_2_-consumption. Moreover, DAAO is supposed to be safe for human consumption, since it is normally expressed in human tissues among which the small intestine (Pollegioni et al., 2007; Sasabe et al., 2016; Sacchi et al., 2018). In addition, it has also been already demonstrated that DAAO alone shows no cytotoxicity against human cells (Rosini et al., 2009).

In conclusion, these results combined with the request from consumers to replace chemical antioxidants and oxygen scavengers with natural compounds, making DAAO an ideal candidate in food preservation.

## Statistics

All experiments were replicated three times. Mean and standard deviation (SD) were calculated using Microsoft Excel 2003 (Microsoft Co., Redmond, WA, United States). One-way analysis of variance was performed using Origin_7.0 SR0 (Origin lab Co., Northampton, MA, USA). Significant effects of treatments were estimated (*p* < 0.05, *p* < 0.01, and *p* < 0.0001).

## Data Availability Statement

All datasets generated for this study are included in the article.

## Author Contributions

GM and LP conceived the experiments and interpreted the results. EB, MA, and GM carried out and interpreted the microbiology experiments. ER determined D-amino acid content in foods. GM and LP wrote the manuscript. All authors reviewed and approved the final manuscript.

### Conflict of Interest

The authors declare that the research was conducted in the absence of any commercial or financial relationships that could be construed as a potential conflict of interest.
